# Hydroxychloride zinc and copper supplementation improves growth, feed efficiency, and gut health in broiler chickens

**DOI:** 10.1038/s41598-025-17713-8

**Published:** 2025-08-29

**Authors:** Vahideh Shay Sadr, Hoai Thi Thanh Nguyen, Lane Pineda, Yanming Han, Reza Barekatain, Mehdi Toghyani

**Affiliations:** 1 School of Life and Environmental Sciences, Faculty of Science, The University of Sydney, Sydney, NSW 2006 Australia; 2 Department of Animal Science, School of Environmental and Rural Science, University of New England, Armidale, NSW 2351 Australia; 3 Trouw Nutrition, Amersfoort, The Netherlands; 4 Sydney School of Veterinary Science, Faculty of Science, The University of Sydney, Sydney, NSW 2006 Australia

**Keywords:** Broiler chicken, Hydroxychloride trace minerals, Gut health biomarkers, Growth promotors, Tibia breaking strength, Cecal microbiota, Biotechnology, Physiology, Zoology

## Abstract

High-dose copper (Cu) supplementation is used as an alternative to antibiotic growth promoters for improving gut health in broiler chickens. The aim of this study was to evaluate the effects of hydroxychloride Zn (HyZ) and Cu (HyC) at different inclusion levels on productive traits and gut health biomarkers in broiler chickens. A total of 990 Ross 308 broilers were assigned to 55 floor pens (18 birds per pen) and received five dietary treatments as follows: (T1) an inorganic diet (INO) with 15 mg/kg Cu from CuSO₄ and 100 mg/kg of Zn from ZnSO₄; (T2) a hydroxychloride diet with 15 mg/kg of Cu from HyC and 100 mg/kg of Zn from HyZ; (T3 and T4) hydroxychloride diets with 80 mg/kg Zn from HyZ and either 100 (T3) or 150 (T4) mg/kg of Cu from HyC; and (T5) a hydroxychloride diet containing 80 mg/kg Zn from HyZ and Cu from HyC supplied at 200 mg/kg in the starter phase (1–10 days), 100 mg/kg in the grower phase (10–24 days), and 60 mg/kg in the finisher phase (24–35 days). Gut integrity and cecal bacterial populations were assessed on day 21, while carcass composition, liver, tibia and excreta mineral profiles, and tibia strength were evaluated on day 35 of the experiment. Hydroxychloride diets significantly improved body weight by ~ 4% at day 35 compared to INO (*P* < 0.05). Feed conversion ratio improved by 2.5% with T2 over INO, with further improvements in T3 (3.8%) and T4 (3.2%) (*P* < 0.05). Birds on T5 performed similarly to T3 throughout the trial (*P* > 0.05). INO birds had the lowest breast meat yield and the highest abdominal fat percentage (*P* < 0.05). Hydroxychloride diets reduced serum FITC-d levels and cecal *Enterobacteria* counts while increasing tibia breaking strength, Zn, and P levels (*P* < 0.01), and tended (*P* = 0.06) to increase cecal *Bifidobacteria* populations. In conclusion, these results suggest that replacing ZnSO₄ and CuSO₄ with HyZ and HyC has the potential to enhance gut health, body weight gain, and feed efficiency in broiler chickens.

## Introduction

Modern broiler chickens, with their high genetic potential and rapid growth rates, exhibit increased responsiveness to dietary interventions^1^. Consequently, contemporary broiler diets are precisely formulated using advanced nutritional models and ingredients profiling to ensure diets are balanced for over 30 essential nutrients, including energy, amino acids, vitamins, macro minerals, and trace minerals, ensuring optimal growth, health, and productivity^2^. Trace minerals, particularly zinc (Zn) and copper (Cu) are essential components of broiler chickens’ diet due to their critical roles in physiological and metabolic processes, including growth, immune function, and antioxidant immune system^3,4^. Zn is a cofactor for numerous enzymes involved in protein synthesis, nucleic acid metabolism, and cell division^5^. Similarly, Cu plays a vital role in iron metabolism, connective tissue formation, and immune function^6,7^. Furthermore, high-dose Cu supplementation, beyond nutritional requirements, has commercially been used as an alternative to antibiotic growth promoters due to Cu antimicrobial properties and positive effects on gut health^8,9^. Cu super dosing has been demonstrated to enhance the intestinal structure and function while positively influencing the gut microbiota composition^10^. Copper’s bactericidal and bacteriostatic properties have been widely documented, showing its ability to modulate the microbial environment within the gastrointestinal tract^11^.

Traditionally, these minerals are supplemented in the form of inorganic salts, such as sulfates and oxides, which, despite their widespread use, are associated with poor bioavailability and undesirable interactions with other dietary components^12,13^. Therefore, Zn and Cu provided as inorganic salts can interact antagonistically with other minerals, such as Fe, as well as with vitamins, enzymes, and even phytate^14^. These interactions may reduce the bioavailability and utilization of both minerals and their antagonistic counterparts in poultry diets^15^. In addition, due to the lower bioavailability of inorganic minerals and their relatively low cost, higher quantities are often included in feed formulations to prevent trace mineral deficiencies^16^. However, this approach can lead to excessive mineral excretion through litter and manure, which poses significant environmental risks by potentially contaminating soil and water resources^17^. To address these limitations, organic sources of Zn and Cu, typically chelated to carbon-containing molecules such as amino acids, have been proposed as effective alternatives to inorganic forms and have been extensively studied^18^. However, their higher cost currently limits their widespread adoption and integration within the poultry industry^19^.

Hydroxychloride trace minerals, a relatively new class of trace mineral sources for livestock, comprise covalent bonds with hydroxyl groups and chloride ions within a stable crystalline matrix, making them more structurally robust than traditional ionic forms^20,21^. This unique structure reduces water solubility compared to ionic counterparts, thereby minimizing the risk of antagonistic interactions in both feed and the upper digestive tract^20^. Previous studies have demonstrated that feeding equivalent levels of Zn from Zn hydroxychloride (HyZ), compared to Zn sulfate, results in significant improvements in growth performance, meat yield, and bone-breaking strength in broiler chickens^21,22^. Similarly, Cu hydroxychloride (HyC) has shown greater efficacy than Cu sulfate in enhancing growth performance, both at nutritional and growth-promoting levels^9^. However, while HyC promotes growth, increasing its dietary concentration from 50 to 200 mg/kg has been associated with a linear increase in Cu excretion^23^.

Despite previous findings on individual trace minerals, limited research has investigated the simultaneous use of HyC and HyZ as complete replacements for their inorganic counterparts in broiler diets. Furthermore, this study is among the first to evaluate a titration approach to HyC supplementation by progressively reducing dietary Cu across feeding phases, as a practical strategy to maintain growth performance while minimizing feed costs and environmental Cu excretion. Therefore, this study aimed to evaluate the effects of replacing inorganic Zn and Cu salts with hydroxychloride forms, provided at nutritional levels, on growth performance, carcass traits, gut health biomarkers, and tissue mineral accumulation in broiler chickens; and to compare the growth-promoting efficacy of continuous versus phased (titrated) HyC supplementation to assess its impact on feed efficiency and Cu excretion.

## Materials and methods

### Ethics approval

All experimental procedures in this study were reviewed and approved by the University of New England Animal Ethics Committee (Approval Number: AEC18-070) and conducted in accordance with national and international guidelines. In accordance with the AVMA 2020 euthanasia guidelines, the birds were first electrically stunned and sedated before being euthanized. Euthanasia was performed through decapitation using a sharp knife to ensure a humane and effective process. Furthermore, this study adheres to the ARRIVE guidelines to ensure the ethical treatment of animals and the transparent reporting of all experimental procedures.

### Experimental design and bird allocation

A total of 990 male day-old Ross 308 chicks were sourced from a commercial hatchery (Darwalla Poultry Distributors Pty Ltd., Mount Cotton, Queensland, Australia) and delivered to the Centre for Animal Research and Training at the University of New England, Armidale, NSW 2351, Australia. Upon arrival, the chicks were weighed and randomly allocated to five dietary treatments using a completely randomized design. Each treatment was replicated 11 times, with 18 chicks housed per floor pen (average initial pen weight: 711 ± 3.0 g).

The main feed ingredients used in this study, wheat, corn, soybean meal, canola meal, and canola oil, were sourced from Nutrien Ag Solutions (6 Endeavour Drive, Armidale, NSW 2350, Australia). As outlined in Table 1, the basal wheat-corn soybean meal diets were formulated to meet the nutrient requirements of the birds, following the breeder recommendations^1^. The diets were offered in three phases: starter (0–10 days), grower (10–24 days), and finisher (24–35 days). The five dietary treatments were designed as follows: T1 (Inorganic diet - INO): 100 mg/kg Zn from ZnSO₄ and 15 mg/kg Cu from CuSO₄; T2 (Hydroxychloride diet): 100 mg/kg Zn from hydroxychloride Zn (HyZ) and 15 mg/kg Cu from hydroxychloride Cu (HyC); T3: 80 mg/kg Zn from HyZ and 100 mg/kg Cu from HyC; T4: 80 mg/kg Zn from HyZ and 150 mg/kg Cu from HyC; T5: 80 mg/kg Zn from HyZ and phase-specific Cu levels of 200 mg/kg for starter, 100 mg/kg for grower, and 60 mg/kg for finisher from HyC. A HyZ dose of 80 mg/kg was selected based on our previous studies, which demonstrated that Zn supplemented in hydroxychloride form at this level is sufficient to optimise growth performance and bone strength in broiler chickens^21,22^. All mineral premixes were prepared at the University of New England nutrition laboratory and analyzed for mineral content before incorporating them into the experimental diets. The Cu and Zn sources used were either feed-grade CuSO₄ and ZnSO₄ or hydroxychloride Cu and Zn (Selko IntelliBond Zn, Trouw Nutrition, Netherlands).

All diets were pelleted, with the starter diet further crumbled to enhance feed intake during the early growth phase. The initial shed temperature was maintained at 34 ± 1.0 °C for the first three days and was gradually reduced to 23 °C by day 21. The lighting program and ventilation were managed in accordance with the recommendations outlined in the Ross 308 breed management manual^24^. Birds had *ad libitum* access to both water and feed throughout the entire study period.

The body weight and feed intake of birds were recorded on a pen basis at day 0 and at the end of each feeding phase to calculate feed conversion ratio (FCR) and body weight gain. The body weight of any dead or culled birds was also recorded and used to adjust FCR calculations. On day 35, a body weight-corrected FCR was calculated and reported to account for treatment-associated differences in body weight. This correction was based on the assumption that a 50 g difference in body weight corresponds to a 1-point change in FCR. The rationale for this adjustment is to reflect commercial growing practices, where birds are typically reared to a target weight rather than a fixed age.

### Sample collection

Triple representative composite samples were collected from all diets and premixes and analyzed in duplicate to determine mineral concentrations. On day 21 of the trial, three birds per pen, selected based on proximity to the mean body weight of the pen, were orally gavaged with fluorescein isothiocyanate-dextran (FITC-d) at a dosage of 4.16 mg FITC-d per kg live body weight (Sigma–Aldrich Co., Castle Hill, NSW, Australia). After 210 min, blood samples were collected from these birds into vacutainers containing lithium heparin for serum separation. Serum samples were harvested into individual Eppendorf tubes and frozen at −20 °C for subsequent FITC-d analysis. The three gavaged birds were euthanized in accordance with the AVMA 2020 euthanasia guidelines, where the birds were first electrically stunned and sedated before being euthanized. Euthanasia was performed through decapitation using a sharp knife to ensure a humane and effective process. Approximately 1 cm sections of the mid-duodenum from each bird were collected, washed with PBS, and fixed in 10% buffered formalin for histomorphology measurements. Following euthanasia, sub-samples of caecal digesta were collected, snap-frozen in liquid nitrogen, and stored at −80 °C for subsequent microbial population analysis using real-time quantitative PCR (qPCR).

On day 35 of the trial, three birds per replicate pen (33 birds per treatment) with body weights close to the pen average were selected and euthanized using the same method described previously. Liver samples were collected individually into 60 mL plastic containers and stored at −20 °C for subsequent mineral analysis.

Additionally, the right tibias were excised from these birds, and all soft tissues and cartilage were carefully removed. Breast fillets, thigh + drumstick, and abdominal fat pads were dissected, weighed, and expressed as a percentage of live body weight (g/100 g) to calculate yield percentages.

Fresh excreta samples were also collected from three birds and pooled per replicate pen. The excreta samples were mixed properly, oven-dried and then ground to pass through 0.5 mm sieve. Sub-samples of the ground excreta were used for mineral analysis.

On the same day, all birds in each pen underwent visual inspections for footpad dermatitis and hock burns. Both feet were examined, and any signs of footpad dermatitis were scored on a scale from 0 to 4, based on the criteria outlined by Stracke et al.^25^. Hock burns were similarly scored from 0 to 4, following the guidelines provided by Welfare Quality^26^.

**Table 1:** Experimental diet composition and calculated nutrient specifications.

Ingredients %	Starter	Grower	Finisher
Wheat	35.2	35.5	34.3
Soybean meal	29.0	24.9	18.9
Corn	25.0	25.0	30.0
Canola meal	5.00	8.00	10.0
Canola oil	2.29	3.51	4.07
Limestone	1.13	1.07	1.01
Dicalcium phosphate 18P/21Ca	0.913	0.764	0.649
Sodium bicarbonate	0.306	0.263	0.177
L-lysine	0.302	0.235	0.222
DL-methionine	0.260	0.218	0.181
Salt	0.182	0.208	0.214
L-threonine	0.149	0.091	0.066
Choline Chloride 60%	0.105	0.096	0.098
Trace mineral premix	0.100	0.100	0.100
Vitamin premix ^1^	0.090	0.090	0.090
Xylanase ^2^	0.020	0.020	0.020
Phytase ^3^	0.010	0.010	0.010
***Calculated nutrients***			
Metabolizable energy (kcal/kg)	3,000	3,080	3,150
Crude protein %	22.62	21.71	19.86
Crude fat %	4.29	5.571	6.29
Crude fiber %	2.83	2.91	2.94
Digestible Arginine%	1.302	1.230	1.090
Digestible Lysine %	1.260	1.150	1.020
Digestible Methionine%	0.580	0.533	0.480
Digestible Methionine + Cysteine %	0.920	0.870	0.800
Digestible Tryptophan %	0.259	0.245	0.216
Digestible Isoleucine %	0.840	0.796	0.710
Digestible Threonine%	0.860	0.770	0.680
Digestible Valine %	0.922	0.886	0.808
Calcium %	0.900	0.850	0.800
Available phosphorus %	0.450	0.425	0.400
Total phosphorus %	0.570	0.552	0.528
Sodium %	0.200	0.200	0.180
Chloride %	0.230	0.230	0.230
Choline mg/kg	1,700	1,600	1,500

^1^Vitamin concentrate supplied per kilogram of diet: retinol, 12,000 IU; cholecalciferol, 5000 IU; tocopheryl acetate, 75 mg, menadione, 3 mg; thiamine, 3 mg; riboflavin, 8 mg; niacin, 55 mg; pantothenate, 13 mg; pyridoxine, 5 mg; folate, 2 mg; cyanocobalamin, 16 µg; biotin, 200 µg; cereal-based carrier, 149 mg; mineral oil, 2.5 mg.

^2^ Xylanase at 200 g/tonne provided approximately 200 fungal xylanase units (FXU) per kilogram of feed.

^3^ Phytase at 100 g/tonne provided approximately 1000 phytase unit (FTU) per kilogram of feed.

### Tibia breaking strength and mineral analysis

The tibias were subjected to a breaking strength test using an Instron instrument (LX 300 Instron Universal Testing Machine, Instron Corp., Canton, USA), equipped with a 30 kN load cell and a three-point fixture bed. The test was conducted at a speed capturing 10 data points per second, and the system was operated via Blue Hill 3 software.

Following the breaking strength measurement, the tibia samples were dried in a Qualtex Universal Series 2000 drying oven (Watson Victor Ltd., Perth, Australia) at 105 °C for 24 h to determine their dry matter content. The dried tibias were then ashed in a Carbolite CWF 1200 chamber furnace (Carbolite, Sheffield, UK) at 600 °C for 6 h, with a ramp-up time of 1 h starting at 300 °C. The moisture-free tibia ash was calculated and expressed as a percentage of the dry tibia weight.

Liver samples were freeze-dried at −50 °C and ground to pass through a 0.5 mm sieve. The mineral content of premixes, feed samples, excreta, liver, and tibia ash were analyzed using an inductively coupled plasma-optical emission spectrometer (ICP-OES, Agilent, Mulgrave, Victoria, Australia). Approximately 0.1 g of each ash sample was weighed into Teflon tubes (Milestone, Sorisole, Italy) and digested using 1 mL distilled water and 4 mL concentrated HCl (70%) in an Ultrawave Microwave Digestion system (Milestone, Sorisole, Italy) for 45 min. After digestion, the solution was cooled to room temperature and quantitatively transferred into a 30 mL volumetric flask. The final volume was adjusted to 25 mL with distilled water and thoroughly mixed. The prepared solutions were then analyzed for trace mineral concentrations using the ICP-OES instrument.

### Serum FITC-d measurement

Serum samples were diluted 1:1 with phosphate-buffered saline (PBS) for the assay, and black 96-well plates were used to minimize crosstalk between samples. The concentration of fluorescein isothiocyanate-dextran (FITC-d) per mL of serum was determined using a SpectraMax M2e Microplate Reader (Molecular Devices, San Jose, California, USA) at an excitation wavelength of 485 nm and an emission wavelength of 528 nm. Fluorescence levels in the samples were converted to FITC-d concentrations (µg/mL of serum) using a standard curve calculated from known FITC-d concentrations.

### Duodenal morphology

The morphological analysis was performed following the method described by M’Sadeq et al.^27^. Duodenal samples were fixed in 10% neutral buffered formalin and processed using paraffin embedding techniques. Tissue samples were prepared in a Leica TP1020 tissue processor (GMI Inc., Ramsey, MN) with the following program: 30% ethanol for 2 h; 50% ethanol for 2 h; 70% ethanol for 2 h; 80% ethanol for 2 h; 95% ethanol for 1 h; absolute ethanol for 1 h (repeated twice); 50:50 ethanol: xylol for 1 h; xylol for 1 h (repeated twice); paraplast for 2 h; and paraplast under vacuum for 2 h.

Sections of 5 μm were cut using a microtome (Leitz 1516; Leica Microsystems, Bensheim, Germany) and mounted on glass slides. Slides were stained using Harris’s hematoxylin and eosin staining method. Specimens were examined using an Olympus CX41 light microscope, and images were captured with Analysis 5.0 software (Olympus Soft Imaging Solutions GmbH, Münster, Germany). Multiple measurements of villus height and crypt depth were taken per replicate and averaged to ensure representative morphological analysis.

### Quantification of caecal bacterial groups

Cecal bacterial DNA extraction was performed as described by Kheravii et al.^28^. Briefly, 65 mg of frozen cecal samples were combined with 300 mg of glass beads, and DNA was extracted using QIAxtractor DNA Reagents and QIAxtractor DNA plasticware kits (Qiagen, Inc., Doncaster, VIC, Australia). Samples were lysed with 300 µL of Qiagen Lysis Buffer, and cells were disrupted using a bead beater mill (Retsch GmbH & Co, Haan, Germany). Subsequently, the samples were incubated at 55 °C for 2 h in a heating block and centrifuged at 20,000 × g for 5 min. DNA was extracted using an X-tractor Gene automated DNA extraction system (Corbett Life Science, Sydney, Australia). The quantity and purity of extracted DNA were assessed using a NanoDrop ND-8000 spectrophotometer (Thermo Fisher Scientific, Waltham, USA). Samples with A260/A280 ratios greater than 1.8 were deemed of high purity and stored at −20 °C for further analysis.

The extracted DNA was diluted 20-fold in nuclease-free water, and a quantitative real-time polymerase chain reaction (qPCR) was performed to quantify seven bacterial groups using a Rotorgene 6000 real-time PCR system (Corbett, Sydney, Australia). Each sample was analyzed in duplicate using an SYBRGreen-based mix (SensiMix SYBR No-Rox, Bioline, Sydney, Australia). The quantified bacterial groups included Total Bacteria, *Bacillus*,* Bacteroides*,* Bifidobacterium*,* Lactobacillus*,* Ruminococcus*, and *Enterobacteria*. Primer sequences used for bacterial quantification were as described by Nguyen et al.^23^. Bacterial populations were expressed as log_10_ (genomic DNA copy number)/g of wet digesta.

### Statistical analysis

All the data derived were checked for normal distribution prior to conducting statistical analysis and then analyzed as one-way ANOVA using the General Linear Model procedure of the JMP (JMP Pro 15). Each single pen was considered as an experimental unit and the values presented in the tables are means with pooled standard error of the mean (SEM) (*n* = 55). When a significant effect of treatment was detected (*P* ≤ 0.05), the means were separated by Tukey’s test.

## Results

### Mineral recoveries

As shown in Tables 2 and 3, the measured Zn and Cu concentrations in the premixes closely aligned with the calculated values, confirming that the targeted Zn and Cu levels were likely achieved. Additionally, the mineral profiles of the experimental diets were consistent with the calculated and intended levels, indicating accurate formulation and proper mixability of the diets and premixes.

**Table 2:** Measured mineral composition of the experimental premixes.

Premixes	g mineral/kg premix			
	Cu	Zn	Mn	Fe
INO Zn 100 mg/kg and Cu 15 mg/kg	16.5	103	85.5	43.9
HyZ 100 mg/kg and HyC 15 mg/kg	16.3	104	85.7	42.3
HyZ 80 mg/kg and HyC 100 mg/kg	104.7	83	86.1	41.9
HyZ 80 mg/kg and HyC 150 mg/kg	155.1	81	83.7	41.9
HyZ 80 mg/kg and HyC 60 mg/kg	63.7	80	84.5	43.0
HyZ 80 mg/kg and HyC 200 mg/kg	204.8	80	83.7	41.2

**Table 3:** Measured mineral composition of the experimental diets for different phases.

Diets	Mineral concentration					
	Cu mg/kg	Zn mg/g	Mn mg/kg	Fe mg/kg	Ca g/kg	*P* g/kg
**Starter diets**						
T1-INO Zn 100 mg/kg and Cu 15 mg/kg	28.3	138	148	178	8.6	6.7
T2-HyZ 100 mg/kg and HyC 15 mg/kg	27.5	141	144	171	8.8	6.6
T3-HyZ 80 mg/kg and HyC 100 mg/kg	112	118	146	175	8.3	6.7
T4-HyZ 80 mg/kg and HyC 150 mg/kg	168	122	145	170	8.5	6.9
T5-HyZ 80 mg/kg and HyC 200 mg/kg	221	116	151	166	8.6	6.8
**Grower diets**						
T1-INO Zn 100 mg/kg and Cu 15 mg/kg	26.5	137	134	168	7.9	6.4
T2-HyZ 100 mg/kg and HyC 15 mg/kg	25.8	134	141	164	7.6	6.5
T3-HyZ 80 mg/kg and HyC 100 mg/kg	107	119	136	157	7.4	6.3
T4-HyZ 80 mg/kg and HyC 150 mg/kg	163	117	134	159	7.6	6.0
T5-HyZ 80 mg/kg and HyC 100 mg/kg	110	120	147	163	7.2	6.2
**Finisher diets**						
T1-INO Zn 100 mg/kg and Cu 15 mg/kg	23.1	129	134	154	6.8	5.8
T2-HyZ 100 mg/kg and HyC 15 mg/kg	24.4	132	136	149	7.1	5.6
T3-HyZ 80 mg/kg and HyC 100 mg/kg	111	111	142	154	7.2	6.0
T4-HyZ 80 mg/kg and HyC 150 mg/kg	157	113	138	142	6.7	5.8
T5-HyZ 80 mg/kg and HyC 60 mg/kg	72.3	118	133	157	7.3	5.9

### Growth performance

Table 4 summarizes the effect of dietary treatments on growth performance parameters over different phases and the entire production phase of 1 to 35 days. There was a significant effect of mineral source on the body weight (BW) of the birds at the end of each production phase (*P* < 0.01). Regardless of HyC dose birds in hydroxychloride groups had higher BW than the INO group on days 10, 24, and 35 of age. Body weight gain followed the same trend, except for the finisher period (24–35 days) where the values observed did not differ statistically among treatments (*P* > 0.05). During the starter and finisher periods (1–10, 24–35 days) INO birds had the highest FCR compared to hydroxychloride birds (*P* < 0.01). However, in the grower phase, there was a further improvement of FCR (1.316 vs. 1.286 and 1.279) by the inclusion of 100 mg/kg HyC compared to hydroxychloride treatment with HyC at 15 mg/kg (T2).

**Table 4:** Effect of experimental diets on performance parameters of broilers over different growth periods. Mean values are based on 18 birds per replicate and 11 replicates per treatment. ^a–c^ values in a row with no common superscripts differ significantly (*P* ≤ 0.05) – Tukey test; ^1^FCR values corrected to the mean body weight gain (1–35 d) of INO group.

Treatments	T1 INO	T2 HyZ 100 HyC 15	T3 HyZ 80 HyC 100	T4 HyZ 80 HyC 150	T5 HyZ 80 HyC 200, 100 & 60	SEM	*P*- value
**Body weight g/b**							
Day 1	39.5	39.6	39.4	39.7	39.2	0.233	0.575
Day 10	307 ^b^	323 ^a^	327 ^a^	323 ^a^	329 ^a^	2.76	0.001
Day 24	1328 ^b^	1396 ^a^	1405 ^a^	1393 ^a^	1422 ^a^	7.44	0.001
Day 35	2557 ^b^	2641 ^a^	2692 ^a^	2651 ^a^	2669 ^a^	15.76	0.001
**Body weight gain g/b**							
1–10 d	268 ^b^	284 ^a^	288 ^a^	283 ^a^	290 ^a^	2.74	0.001
10–24 d	1021 ^b^	1073 ^a^	1078 ^a^	1070 ^a^	1092 ^a^	7.06	0.001
24–35 d	1229	1244	1287	1258	1247	17.27	0.198
1–35 d	2518 ^b^	2601 ^a^	2654 ^a^	2611 ^a^	2630 ^a^	15.76	0.001
**Feed intake g/b**							
1–10 d	293	299	302	297	302	3.13	0.246
10–24 d	1373	1411	1386	1392	1398	10.33	0.137
24–35 d	1997	1979	2024	1990	1955	28.98	0.560
1–35 d	3665	3691	3713	3680	3656	28.27	0.642
**FCR g/g**							
1–10 d	1.095 ^a^	1.055 ^b^	1.049 ^b^	1.051 ^b^	1.040 ^b^	0.004	0.001
10–24 d	1.346 ^a^	1.316 ^b^	1.286 ^bc^	1.302 ^bc^	1.279 ^c^	0.006	0.001
24–35 d	1.625 ^a^	1.591 ^b^	1.572 ^b^	1.581 ^b^	1.568 ^b^	0.007	0.001
1–35 d	1.455 ^a^	1.419 ^b^	1.399 ^cd^	1.409 ^bc^	1.390 ^d^	0.005	0.001
BWc FCR^1^	1.455 ^a^	1.402 ^b^	1.372 ^cd^	1.391 ^bc^	1.367 ^d^	0.005	0.001
**Liveability %**							
1–35 d	96.9	97.5	96.0	98.0	98.5	0.958	0.402

Considering the entire production period (1–35 d) FCR significantly improved by 2.5% with T2 over INO (*P* < 0.01), with further improvements in T3 (3.8%) and T4 (3.2%) in response to higher HyC. When FCR values were corrected to the body weight of the INO group, the improvement in FCR in response to HyZ and HyC were 5.3, 8.3, and 6.4 points for T2, T3, and T4, respectively compared to INO treatment (*P* < 0.05).

Inclusion of 200, 100, and 60 mg/kg of HyC in starter, grower, and finisher diets, respectively, resulted in similar BW and FCR to 100 mg/kg HyC throughout the entire production period (*P* > 0.05) and improved BW corrected FCR by 8.8 and 3.5 points compared to INO and T2 groups, respectively (*P* < 0.05). No significant effect of dietary treatment was observed on feed consumption and mortality rate (*P* > 0.05).

### Carcass characteristics, foot pad lesions, and serum FITC-d

The lowest breast meat yield and highest abdominal fat percentage were recorded in birds fed the INO diets (Table 5; *P* < 0.05). Thigh + drumstick, liver, and gizzard weights remained unaffected by the dietary treatments (*P* > 0.05).

**Table 5:** Carcass characteristics (g/100 g live weight), foot pad and Hock lesions, and serum FITC-d levels in response to dietary treatments. Mean values are based on 3 birds per replicate and 11 replicates per treatment; ^a–b^ values in a row with no common superscripts differ significantly (*P* ≤ 0.05) – Tukey test.

Treatments	T1 INO	T2 HyZ 100 HyC 15	T3 HyZ 80 HyC 100	T4 HyZ 80 HyC 150	T5 HyZ 80 HyC 200, 100 & 60	SEM	*P*- value
Breast	18.7 ^b^	19.4 ^a^	19.5 ^a^	19.5 ^a^	19.7 ^a^	0.154	0.001
Thigh + drumstick	19.3	19.6	19.5	19.1	19.2	0.215	0.543
Fat pad	1.11 ^a^	0.96 ^b^	0.95 ^b^	0.98 ^b^	0.97 ^b^	0.040	0.039
Liver	2.57	2.52	2.57	2.43	2.52	0.076	0.715
Gizzard	1.77	1.82	1.77	1.74	1.64	0.048	0.152
Footpad dermatitis	0.91	0.91	0.82	0.82	0.72	0.293	0.991
Hock burn	1.91	1.36	1.63	1.82	0.91	0.257	0.058
FITC-d (µg/mL)	0.232^a^	0.206^b^	0.207^b^	0.205^b^	0.209^b^	0.006	0.025

Footpad dermatitis and hock burn scores were not significantly affected by treatments (*P* > 0.05), but birds in HyC 200 to 60 mg/kg (T5) tended (*P* = 0.058) to have the lowest hock burn scores compared to the rest of the treatments. The highest FITC-d concertation in serum was measured in the INO group compared to all the hydroxychloride groups (*P* < 0.05).

### Tibia mineral profile and breaking strength

According to the data presented in Table 6, tibia P % in T2 (HyZ 100 mg/kg and HyC 15 mg/kg) was significantly higher than the other treatments (*P* < 0.05). There was also a tendency (*P* = 0.097) for higher Cu levels being retained in tibia samples from HyZ 80 mg/kg and HyC 150 mg/kg treatment. The lowest and highest tibia Zn concentrations were observed in the INO and hydroxychloride groups, respectively (*P* < 0.05). There was a significant effect of treatments on tibia-breaking strength (*P* < 0.01), where hydroxychloride treatments resulted in the highest values compared to the INO group.

**Table 6:** Tibia mineral profile and breaking strength (BS) determined on day 35 in response to dietary treatments. Mean values are based on 3 birds per replicate and 11 replicates per treatment. ^a–c^ values in a row with no common superscripts differ significantly (*P* ≤ 0.05) – Tukey test.

Treatments	T1 INO	T2 HyZ 100 HyC 15	T3 HyZ 80 HyC 100	T4 HyZ 80 HyC 150	T5 HyZ 80 HyC 200, 100 & 60	SEM	*P*- value
Ash (%)	48.1	48.1	48.9	48.3	48.2	0.407	0.620
Ca (%)	38.1	38.5	38.2	39.1	38.2	0.413	0.438
P (%)	17.9^b^	18.4^a^	17.6^b^	18.1^ab^	17.8^b^	0.131	0.001
Cu (µg/g)	2.76	2.89	2.64	3.14	2.67	0.140	0.097
Mn (µg/g)	12.8	13.0	12.4	12.7	13.3	0.389	0.607
Fe (µg/g)	340	363	323	329	337	31.69	0.915
Zn (µg/g)	443^b^	489^a^	461^b^	470^ab^	464^ab^	16.93	0.007
Tibia BS (N/g)	123 ^c^	138 ^b^	152 ^a^	135 ^b^	140 ^b^	4.92	0.004

### Liver and excreta mineral concentration

The mineral composition of the livers is presented in Table 7. Liver content of Ca, P, Cu, Zn, and Mn were not significantly (*P* > 0.05) affected by dietary treatments. However, Zn and Cu concentrations were numerically higher in hydroxychloride groups compared to INO treatment. The highest and lowest Fe concentration was recorded in T4 (HyZ 80 mg/kg and HyC 150 mg/kg) and INO treatments, respectively (*P* < 0.05).

**Table 7:** Liver and excreta mineral concentration determined on day 35. Mean values are based on 3 birds per replicate and 11 replicates per treatment. ^a–d^ values in a column with no common superscripts differ significantly (*P* ≤ 0.05) – Tukey test.

Treatments	Ca (g/kg)		*P* (g/kg)		Cu (mg/kg)		Mn (mg/kg)		Fe (mg/kg)		Zn (mg/kg)	
	Liver	Excreta	Liver	Excreta	Liver	Excreta	Liver	Excreta	Liver	Excreta	Liver	Excreta
**T1** **INO**	0.216	11.3	11.0	8.31 ^a^	12.98	72.3 ^d^	10.88	485	503^b^	539 ^a^	86.2	343 ^a^
**T2 HyZ 100** **HyC 15**	0.214	10.6	11.4	7.45 ^b^	14.19	64.6 ^d^	10.92	466	597^ab^	366 ^b^	90.4	257 ^b^
**T3 HyZ 80** **HyC 100**	0.205	11.4	11.1	6.61^b^	13.25	433 ^b^	10.78	474	645^ab^	385^b^	87.8	222^b^
**T4 HyZ 80** **HyC 150**	0.208	9.41	11.2	7.84 ^ab^	13.57	645 ^a^	11.4	491	706^a^	421 ^b^	89.3	231 ^b^
**T5 HyZ 80** **HyC 200**,** 100 & 60**	0.225	11.1	11.0	6.82 ^b^	13.11	256 ^c^	11.57	425	673^ab^	387 ^b^	87.3	264 ^b^
**SEM**	0.006	0.520	0.150	0.382	0.407	22.2	0.348	36.4	48.5	41.9	2.01	18.3
**P- value**	0.298	0.245	0.493	0.041	0.253	0.001	0.404	0.677	0.042	0.015	0.618	0.008

According to the data presented in Table 7, feeding the INO diets significantly increased the concentration of P, Fe, and Zn in excreta compared to the hydroxychloride treatments (*P* < 0.05). The lowest excreta Cu content was measured in INO and T2 (15 mg/kg) groups and increasing the HyC to 100 and 150 mg/kg increased Cu excretion at each level of increase (*P* < 0.01). However, birds in T5 receiving the HyC titration program excreted lower Cu than the 100 and 150 mg/kg of HyC.

### Caecal microbiota populations

As shown in Table 8, *Bifidobacteria* counts tended (*P* = 0.06) to be higher in all hydroxychloride groups compared to the INO group. The inclusion of higher HyC in the diet significantly (*P* < 0.01) reduced caecal *Entrobacteria* counts compared to T2 and INO treatments with 15 mg/kg of Cu. Other bacterial groups were not statistically affected by the dietary treatments.

**Table 8:** Bacterial composition (Log_10_ copy numbers g^−1^) in Caeca content determined through qPCR on day 21. Mean values are based on 3 birds per replicate and 11 replicates per treatment. ^a–c^ values in a row with no common superscripts differ significantly (*P* ≤ 0.05) – Tukey test.

Treatments	T1 INO	T2 HyZ 100 HyC 15	T3 HyZ 80 HyC 100	T4 HyZ 80 HyC 150	T5 HyZ 80 HyC 200, 100 & 60	SEM	*P*- value
*Lactobacillus*	8.56	8.47	8.53	8.49	8.34	0.091	0.500
*Bifidiobacteria*	8.88	9.14	9.16	9.10	9.32	0.103	0.060
*Bacteroids*	6.02	5.94	5.87	5.96	5.97	0.081	0.787
*Bacillus*	8.32	8.11	8.30	8.18	8.10	0.117	0.547
*Ruminococcus*	9.54	9.48	9.51	9.55	9.52	0.049	0.856
*Entrobacteria*	9.36^a^	9.27^ab^	8.84^c^	8.98^bc^	8.91^c^	0.111	0.004
Total Bacteria	10.84	10.73	10.77	10.72	10.73	0.060	0.656

### Duodenum histology

**Fig. 1:**
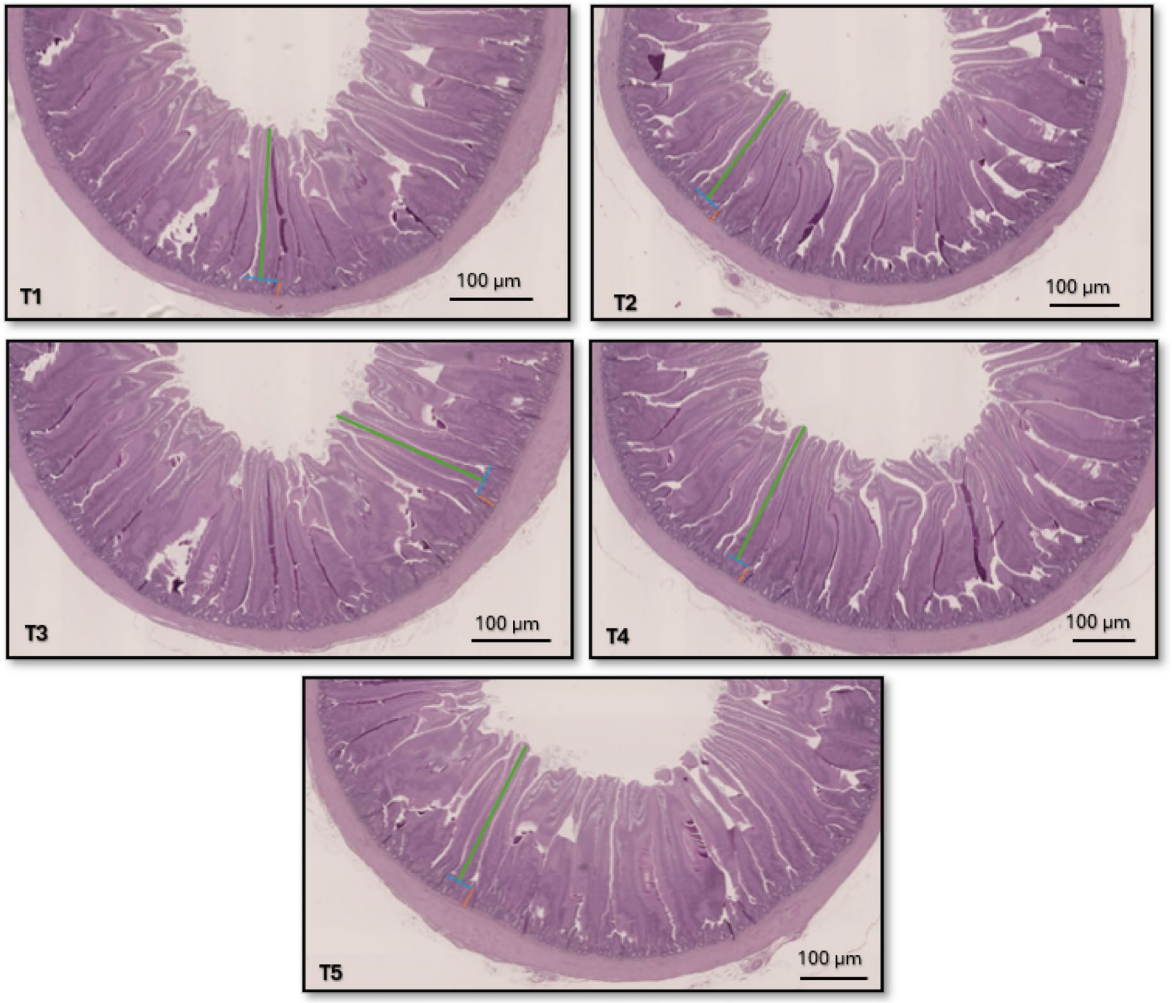
Histological representation of the duodenum of birds on day 21 (scale bar: 100 μm). Villus height (green line) was measured from the tip of the villus to the villus–crypt junction (blue line), and crypt depth was measured as the depth of the invagination between adjacent villi (orange line). Multiple measurements were taken per sample and averaged. Villus height and crypt depth were similar across treatments, indicating no significant effect of diet on duodenal morphology.

Dietary treatments had no significant effect on duodenal villus height (1424–1529 μm), crypt depth (180–187 μm), or the villus height-to-crypt depth ratio (7.9–8.2) (Fig. 1; *P* > 0.05).

## Discussion

Overall, the birds in this study outperformed the breeder guidelines for male broilers, achieving higher body weights, which in turn resulted in increased feed intake^29^. Notably, even the birds in the INO group surpassed the breeder guidelines, with a final body weight of 2557 g compared to the guideline value of 2441 g per bird. However, supplemental Zn and Cu provided in the form of sulphates may still affect the bird’s overall metabolism, potentially leading to reduced feed efficiency, decreased breast meat yield, and increased abdominal fat deposition, as observed in this study.

The significantly higher final body weight and improved feed efficiency observed in all hydroxychloride groups, compared to the INO group, highlight the benefits of providing a more bioavailable source of Zn and Cu to fast-growing broiler chickens. These results suggest that modern broiler chickens not only meet but can exceed their genetic potential when fed with highly bioavailable trace mineral sources. Other studies have reported improved body weight gain and feed efficiency when Zn^22^ and Cu^9^ are supplied in the form of hydroxychloride.

Research has shown that organic or chelated mineral sources with optimal chelation strengths provide higher bioavailability than inorganic sources^30,31^. Historically, this advantage has been attributed to reduced dietary interference and antagonism, allowing these minerals to reach the intestinal brush border, where they are hydrolyzed and absorbed as ions^32^. However, kinetic data from Bai et al.^33^ suggests that the transport of organic minerals with higher chelation strength (quantified as QF) follows a saturable process, indicating the involvement of a distinct transport pathway in the duodenum, separate from the system used for inorganic minerals. The small intestine, particularly the duodenum, is the primary site for the absorption of trace minerals. This absorption occurs via specific transporters that are regulated by the body’s biological requirements and influenced by digestive dynamics, ensuring efficient uptake based on metabolic demands^34,35^. Consequently, the higher growth rate observed in birds fed HyZ and HyC diets may further enhance intestinal mineral uptake and reduce mineral excretion.

Birds fed hydroxychloride-supplemented diets excreted lower levels of P, Zn, and Fe, which aligns with the higher liver Fe concentrations observed in these birds compared to those fed the INO diets. This suggests improved bioavailability and stability of hydroxychloride (HyZ and HyC) sources compared to traditional sulfate-based minerals. For example, the presence of phytate in the upper gastrointestinal tract is known to interfere with trace mineral absorption. Phytate, being negatively charged, readily binds to positively charged minerals like Zn and Cu, forming insoluble complexes that reduce the bioavailability of these minerals as well as phosphorus^36^. Therefore, the improved performance of birds fed the hydroxychloride-supplemented diets could be attributed to their reduced reactivity in the digestive tract, thereby minimizing such antagonistic interactions and enhancing mineral uptake. Furthermore, the lower excretion of P, Zn, and Fe in birds fed hydroxychloride-supplemented diets may be linked to their increased growth rate and larger body frame, which elevate mineral requirements to support greater body mass. P, Zn, and Fe are necessary for normal growth and development in broilers and support proper skeletal and muscular development^37^. In agreement with the findings of this study, Dos Santos et al.^38^ reported that supplementing broiler diets with 100 mg/kg of HyZ and 15 mg/kg of HyC significantly improved tibia characteristics, including higher bone mineral density of the tibia proximal epiphysis, increased tibia ash content, and enhanced overall tibia strength.

Similar to the current findings, a study by Nguyen et al.^23^ showed that increasing dietary Cu in the form of HyC supplementation in 50 mg/kg increments up to 200 mg/kg resulted in a linear increase in Cu excretion in broiler chickens. The titration of HyC in the present study led to lower Cu excretion compared to continuous high-dose HyC supplementation. While Cu excretion increased with higher dietary Cu levels, birds fed the titrated Cu program (200-100-60 mg/kg) exhibited significantly lower Cu excretion than those receiving fixed high Cu levels (100 and 150 mg/kg HyC). Given that finisher feed accounts for nearly 60% of the total feed consumed by birds, titrating Cu inclusion across production phases not only reduces feed costs by lowering HyC supplementation but also mitigates excessive Cu excretion while maintaining growth performance comparable to continuous high HyC feeding throughout the production cycle. The observed reduction in Cu excretion has significant environmental implications, as excessive Cu in poultry manure can accumulate in soil, disrupt microbial communities, and contribute to heavy metal contamination in agricultural systems^39^.

Beyond mineral metabolism, the inclusion of HyC and HyZ had notable effects on gut health and permeability. FITC-d is a widely recognized biomarker for intestinal permeability, with elevated serum levels reflecting compromised epithelial integrity and increased paracellular leakage^40^. Birds fed hydroxychloride diets exhibited lower serum FITC-d concentrations compared to those on the INO diet, indicating improved gut barrier integrity. The lower FITC-d levels suggest that hydroxychloride minerals contribute to tighter intestinal junctions, thereby reducing the translocation of undigested nutrients and microbial toxins into the systemic circulation. Furthermore, the modulation of gut microbiota in response to HyC supplementation provides additional evidence for its role in enhancing gut health. The reduction in cecal *Enterobacteria* counts in response to high HyC dose and the tendency for increased *Bifidobacteria* populations in birds fed hydroxychloride diets suggest a shift toward a more favorable microbial balance. *Enterobacteria*, including pathogenic species such as *Escherichia coli* and *Salmonella*, are associated with intestinal inflammation, compromised gut integrity, and increased permeability^41^. In contrast, *Bifidobacterium* species contribute to gut homeostasis by producing short-chain fatty acids, modulating immune responses, and inhibiting pathogenic colonization^42^. The reduction in *Enterobacteria* populations, coupled with a tendency for increased *Bifidobacterium* counts, may have contributed to the improved intestinal barrier function observed in birds receiving hydroxychloride minerals. The superior bioavailability and lower reactivity of hydroxychloride minerals likely explain their beneficial effects on gut health. Unlike sulfate-based minerals, which readily dissociate in the upper gastrointestinal tract and interact with dietary components, hydroxychloride minerals exhibit greater stability in the digestive environment. This reduces their potential to generate free radicals or interact with other nutrients in ways that may compromise gut health^43^.

The significantly lower abdominal fat pad and higher breast meat yield percentage observed in hydroxychloride-fed birds, compared to the INO group, suggest improved energy partitioning and protein metabolism. This indicates a shift toward lean tissue accretion rather than fat storage. Both Zn and Cu play essential regulatory roles in lipid metabolism, influencing processes such as lipogenesis, lipolysis, and adipocyte differentiation^3^. Furthermore, the higher gut permeability in the INO group, as evidenced by elevated serum FITC-d levels, may have contributed to increased systemic inflammation and oxidative stress. These physiological stressors can divert energy away from muscle accretion and toward immune responses, reducing overall growth efficiency. Additionally, Zn is known to regulate the activity of hormone-sensitive lipase and leptin, modulating lipid mobilization and reducing fat deposition^44^. Cu serves as a cofactor for copper/zinc superoxide dismutase^45^, an antioxidant enzyme that mitigates oxidative stress, a key driver of lipid accumulation in adipose tissues^46^. Beyond lipid metabolism, Zn and Cu are critical for protein synthesis and skeletal muscle development. Zn functions as a vital cofactor for ribosomal activity, while Cu supports enzymatic functions related to oxidative metabolism and connective tissue integrity^4,5^. Therefore, the increased breast meat yield in hydroxychloride-fed birds suggests enhanced protein turnover efficiency, potentially driven by greater Zn and Cu bioavailability leading to improved feed conversion efficiency.

The dietary treatments had no significant effect on foot pad dermatitis and hock burn lesions. Footpad and hock lesions in poultry are primarily influenced by factors such as litter moisture, bedding material, stocking density, and bird activity^47^. Since feed intake was not affected by Zn and Cu sources or levels, it is plausible to suggest that fecal output, and consequently litter quality, was not significantly impacted by dietary treatments to an extent that would influence the occurrence of footpad and hock lesions.

## Conclusion

The findings of this study show that replacing inorganic sulfate-based Zn and Cu with hydroxychloride sources significantly enhances broiler growth performance, feed efficiency, and carcass yield while improving gut health and mineral retention. The superior bioavailability and stability of hydroxychloride minerals facilitate greater Zn and Cu uptake, leading to higher breast meat yield and reduced abdominal fat deposition, indicating improved energy partitioning toward lean tissue accretion. The higher dose of HyC appears to exert an antimicrobial effect, further enhancing feed efficiency, likely through improvements in gut health and microbial balance. Additionally, the titration of HyC (200-100-60 mg/kg) across production phases effectively minimizes excessive Cu excretion while maintaining the performance benefits observed with high-dose HyC supplementation throughout the production cycle, thereby improving environmental sustainability. Future research should further investigate the molecular mechanisms regulating Zn and Cu homeostasis and intestinal integrity in response to hydroxychloride supplementation, optimizing mineral inclusion strategies for sustainable and efficient meat chicken production.

## Data Availability

The datasets used and/or analyzed during the current study are available from the corresponding author on reasonable request.
